# Structural Design of Three-Dimensional Graphene/Nano Filler (Al_2_O_3_, BN, or TiO_2_) Resins and Their Application to Electrically Conductive Adhesives

**DOI:** 10.3390/polym11101713

**Published:** 2019-10-18

**Authors:** Chia-Hsin Zhang, Chia-Hung Huang, Wei-Ren Liu

**Affiliations:** 1 Department of Chemical Engineering, R&D Center for Membrane Technology, Center for Circular Economy, Chung-Yuan Christian University, Chungli 32023, Taiwan; cindy560559@gmail.com; 2 Metal Industries Research and Development Centre, Kaohsiung 81160, Taiwan; chiahung@mail.mirdc.org.tw

**Keywords:** graphene, electrically conductive adhesive, fillers, TiO_2_, Al_2_O_3_, BN, resins

## Abstract

In this study, we designed a three-dimensional structure of electrically conductive adhesives (ECAs) by adding three different kinds of nano filler, including BN, TiO_2_, and Al_2_O_3_ particles, into a few-layered graphene (FLG)/polymer composite to avoid FLG aggregation. Three different lateral sizes of FLG (FLG3, FLG8, and FLG20) were obtained from graphite (G3, G8, and G20) by a green, facile, low-cost, and scalable jet cavitation process. The corresponding characterizations, such as Raman spectroscopy, scanning electron microscopy (SEM), atomic force microscopy (AFM), and transmission electron microscopy (TEM), verified the successful preparation of graphene flakes. Based on the results of four-point probe measurements, FLG20 demonstrated the lowest sheet resistance value of ~0.021 Ω/■. The optimized ECAs’ composition was a 60% solid content of FLG20 with the addition 2 wt.% of Al_2_O_3_. The sheet resistance value was as low as 51.8 Ω/■, which was a reduction of 73% compared to that of pristine FLG/polymer. These results indicate that this method not only paves the way for the cheaper and safer production of graphene, but also holds great potential for applications in energy-related technologies.

## 1. Introduction

The electronic industry is currently one of the most diversified industries. Electronic products can be seen everywhere in daily life, since the new generation of electronic products emphasizes the needs of personalization and portability [1,2]. Among the recent advances in electronic packaging technologies, electrically conductive adhesives (ECAs) have attracted most researchers’ attention. The characteristics of ECAs are that they are environmentally friendly, bendable, have a high workability, and are simple to apply [3,4,5,6,7,8,9,10]. In ECAs, the electrically conducive fillers play a significant role in improving conductivity and strength. Different kinds of electrically conductive fillers, such as silver [11,12,13], copper [14,15], carbon black, carbon nanotubes, graphite, and graphene [16,17,18,19,20,21], have been widely reported. In recent years, carbon-based electrically conductive filler applications in ECAs have been universal because the cost of these materials is lower than that of metal fillers and they demonstrate a much better stability. Among these carbon-based materials, graphene has drawn much attention due to its exceptionally high crystallinity and electronic quality, and these features mean that graphene has high mechanical properties (>1060 GPa), high electrical conductivity (10^4^ S/m), high thermal conductivity (~3000 W/m K), and a light weight [22,23,24]. These unique thermal, mechanical, optical, and electrical properties are better than those of other carbon materials, so this material has been widely used in energy storage materials, lithium ion battery materials, solar cells, super capacitors, and other applications [25,26,27,28].

There are a lot of approaches for preparing graphene-based materials, such as chemical reduction [29], pyrolytic graphite [30], Hummers method [31], and jet cavitation [32,33]. Nevertheless, strong acids, organic solvents, and oxidants are always used in an environmentally unfriendly way during the production process. Therefore, in our study, we propose a jet cavitation-assisted green process to synthesize few-layered graphene (FLG). This method is facile, low cost, green, and scalable to the production of few-layered graphene. Therefore, in the first part of our study, we used FLGs to construct an electrically conductive network in a polymer matrix to increase the electrical conductivity properties of ECAs.

The dispersion of FLGs in a polymer matrix is another important issue. In order to avoid the aggregation of FLGs in a polymer, nano-sized fillers are required to fill spaces between FLGs and the polymer matrix. He et al. [6] investigated graphene/MnO_2_ composite networks as flexible supercapacitor electrodes, lowering the electrical conductivity of the graphene/MnO_2_ composite due to the increase of MnO_2_ with its low electronic conductivity of 10^−5^~10^−6^ S/cm [34]. Pu et al. investigated the application of N-GNSs (N-doped graphene nanosheets) in Ag-filled ECAs to reduce the resistivity with lower Ag loading ratios, and showed that adding merely 1 wt.% of N-GNSs can convert a non-conducting 30 wt.% Ag-filled polymer resin into an ECA with a resistivity of 4.4 × 10^−2^ Ω-cm [7]. Peng et al. advanced the weight ratio of SGNs to silver flakes to 20:80 (%), and the resistivity reached the lowest value of 2.37 × 10^−4^ Ω cm [35]. Ghaleb et al. demonstrated the effect of GNP (Graphene nanoplates) loading (0.05–1 vol%) on the tensile and electrical properties of GNP/epoxy thin-film composites, and the electrical conductivity of the 0.1 vol% GNP thin film increased from 4.32 × 10^−7^ to 1.02 × 10^−3^ S/m [36]. The above’s cost is higher and CNTs also agglomerate easily. In order to overcome the possible drawback of graphene aggregation, we used alumina (Al_2_O_3_), titanium dioxide (TiO_2_), and hexagonal boron nitride (BN) in a graphene/polymer composite. Alumina is abundant in the world and it is low cost, exhibiting characteristics of anti-oxidation, corrosion resistance, and chemical and thermal stability. It not only prevents carbon from being oxidized in the air, but also has a high stability when combined with materials and polymers [37]. Titanium dioxide seemed to be desirable when investigating the electrical properties of semiconducting crystals [38]. In terms of boron nitride, during the conduction process, the efficiency of the electronic product is lowered due to the heat release, so our study investigated the thermal conductivity of boron nitride. These fillers would effectively prevent graphene agglomeration and significantly reduce the cost [39,40].

In our study, we firstly synthesized three different sizes of FLGs by a low-temperature ultra-high pressure continuous homemade flow cell disrupter. Secondly, we incorporated Al_2_O_3_, TiO_2_, and BN particles into the matrix resin to prepare ECAs in order to prevent FLG stacking. The corresponding characterizations, such as Raman spectroscopy, scanning electron microscopy (SEM), atomic force microscopy (AFM), and transmission electron microscopy (TEM), as four-point probe measurements of FLG and FLG/polymer and FLG/fillers/polymer composites, were carried out in this research.

## 2. Experimental Section

### 2.1. Preparation of Few-Layered Graphene (FLG)

First, three different lateral sizes of graphite, including KS-6 (Timcal^®^, d_50_ = 3 μm, namely G3), 8 μm graphite (KNANO^®^, d_50_ = 8 μm, namely G8)), and KS-44 (Timcal^®^, d_50_ = 20 μm, namely G20)), were dispersed in 500 mL of deionized (DI) water by sonication for 15 min (about solid content 5 wt.%). After dispersing them sufficiently, the solution was transferred into the tank of the low-temperature ultra-high pressure continuous homemade flow cell disrupter (LTHPD). As part of the work in designing the LTHPD, the suspension was poured into the device and the process was operated three times at each pressure (800, 1200, and 1500 bar). Therefore, the process was conducted nine times in total. Then, the graphite was separated by cavitation effects with different pressures. The course operated in a circulation cooling water bath, which kept the temperature at 14–16 °C The whole exfoliated experiment was carried out in room conditions. The suspension of graphene was vacuum-filtered to obtain graphene cake and was transferred to an oven at a temperature of 40 °C for 24 h. Finally, the cake of FLG was milled into powder with a grinder.

### 2.2. Preparation of Electrically Conductive Adhesives (ECAs)

The ECAs were mainly composed of a resin matrix, FLG, and nano fillers (Al_2_O_3_, TiO_2_, and BN). First, A-polymer and B-polymer were mixed at the mass ratio of 1:1. Then, ethyl acetate was added to the resin drop by drop over 30 min, with stirring. The graphene and filler samples were added to the resin with various mass ratios, such as 95:5, 90:10, and 85:15, and the total mass fraction was 50%. After stirring for 24 h, the graphene/filler composite was dispersed into the resin. The slurry was coated on the PET (Polyethylene terephthalate) and cured at 150 °C for 2 h in the oven. Finally, the FLG/nano filler content was increased to 55% and 60% in order to improve the ECAs’ efficacy.

### 2.3. Characterizations

The morphologies of the sample were analyzed using scanning electron microscopy (SEM) by Hitachi S-4100 (Tokyo, Japan) and high-resolution transmission electron microscopy (HRTEM) JEOL-JEM2000FXII. The height profile of the as-synthesized few-layer graphene was measured by using atomic force microscopy (AFM, Bruker Dimension Icon, Berlin, Germany). Raman spectra were measured by a micro Raman spectroscopy system (Hsinchu, Taiwan), with a laser frequency of 532 nm as the excitation source. The electrical conductivities of the graphene adhesives were measured using a resistivity meter (KeithLink TG2, Shenzhen, China) with a four-point probe.

## 3. Results and Discussion

**Figure 1:**
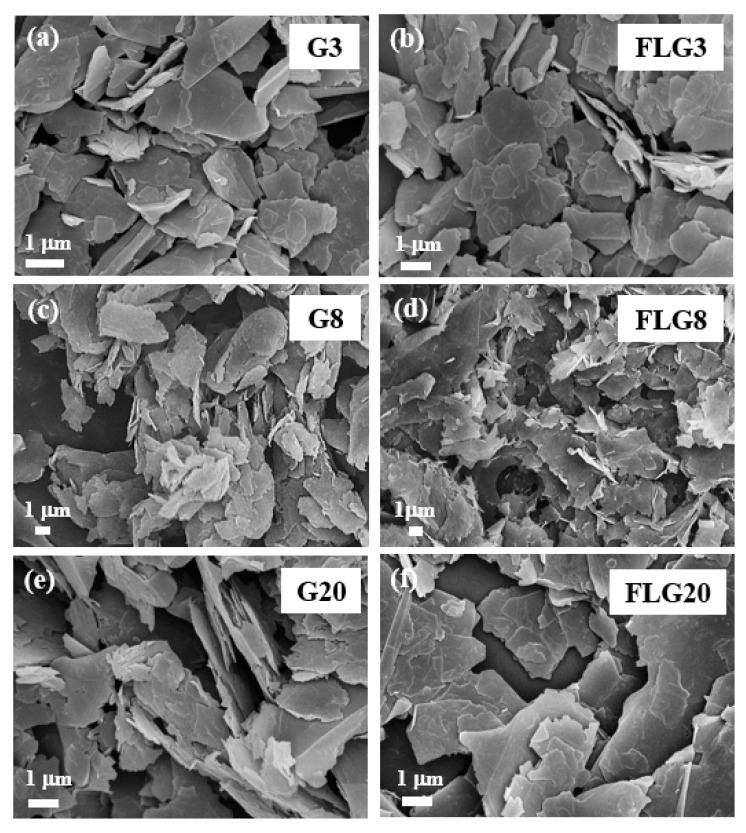
Scanning electron microscopy (SEM) images of (**a**) graphite (G)3, (**b**) few-layered graphene (FLG)3, (**c**) G8, (**d**) FLG8, (**e**) G20, and (**f**) FLG20.

The morphologies of graphite and FLG were observed by SEM (Figure S1a–d in Supplementary Materials). Figure 1a,c,e present the different size graphite images of G3, G8, and G20, which were arranged two-dimensional material and comprised of micron-sized stackable sheet structures, and the lateral sizes of G3, G8, and G20 were in the range of 3–5, 8–10, and 20–22 μm, respectively. These were thus larger and had a greater thickness than FLG. Both FLGs were efficiently exfoliated to form separated thin sheets, as shown in Figure 1b,d,f, demonstrating that FLG3, FLG8, and FLG20 were obtained by LTHPD, respectively, and the lateral sizes were in the range of 2–3, 5–8, and 17–20 μm. In comparison, FLG was composed of thinner sheets and smaller sizes than graphite.

**Figure 2:**
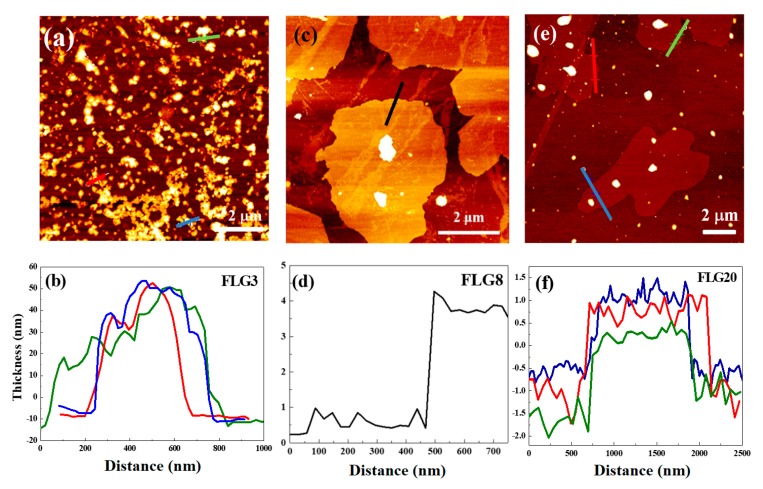
Atomic force microscopy (AFM) images of (**a**) FLG3, (**c**) FLG8, and (**e**) FLG20. Distribution of the thickness of (**b**) FLG3, (**d**) FLG8, and (**f**) FLG20 calculated from the obtained AFM analysis.

We used atomic force microscopy (AFM) to characterize the thickness and surface morphology of as-synthesized FLGs. Figure 2a,c,e depict the AFM images of FLG3, FLG8, and FLG20. Figure 2b,d,f show the thickness which was measured from the height profile of the AFM image, and the average thickness was about 15, 3, and 2 nm, respectively. Since the lateral size of FLG3 was small, the effect of being stripped was poor, and the inverted thickness did not decrease significantly. However, this is consistent with the data reported in the literature, indicating that the thickness of graphene sheets was about 2–4 nm, so FLG8 and FLG20 were high-quality few-layered graphene.

**Figure 3:**
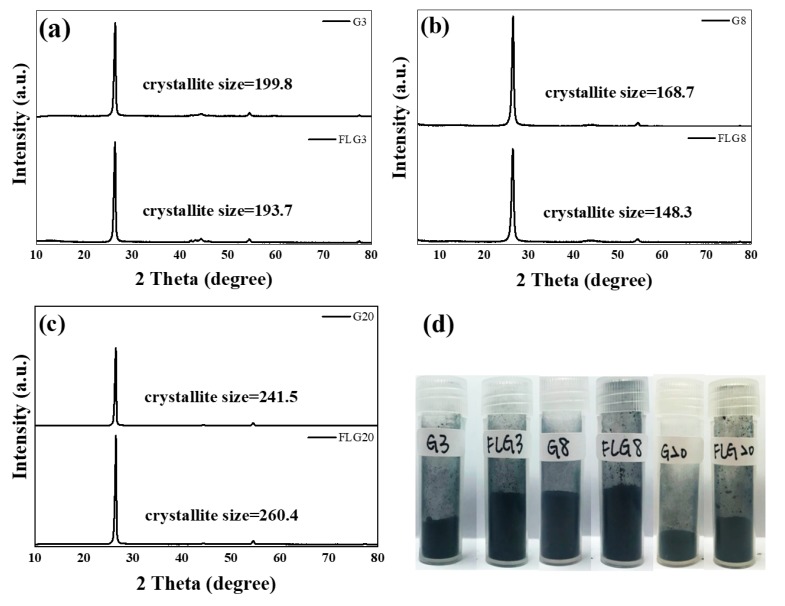
XRD patterns of (**a**) G3 and FLG3, (**b**) G8 and FLG8, and (**c**) G20 and FLG20, and a (**d**) comparison of those of powder before and after manufacturing by a low-temperature ultra-high pressure continuous homemade flow cell disrupter (LTHPD).

Figure 3a–c show the XRD patterns of graphite and FLG. The XRD patterns of graphite indicated the presence of two peaks at 25.0° and 43.5°, which corresponded to the inter-layer spacing of graphite d_002_ and the d_101_ reflection of the carbon atoms, respectively. Additionally, after exfoliating, the intensities of the diffraction peaks present a slight decrease in the few-layered graphene. The average crystallite size of G3, G8, and G20 was about 200,168, and 240 **Å**, respectively. However, the few-layered graphene obtained by LTHPD exhibited a slight decrease in crystallite sizes because the process wrecked the crystallinity. In Figure 3d, the picture shows a comparison of powder before and after manufacturing by LTHPD. The FLG produced by LTHPD was fluffier than that of graphite, so using this feature to prepare ECAs can reduce the amount of graphite and increase the conductive path to improve the conductivity.

**Figure 4:**
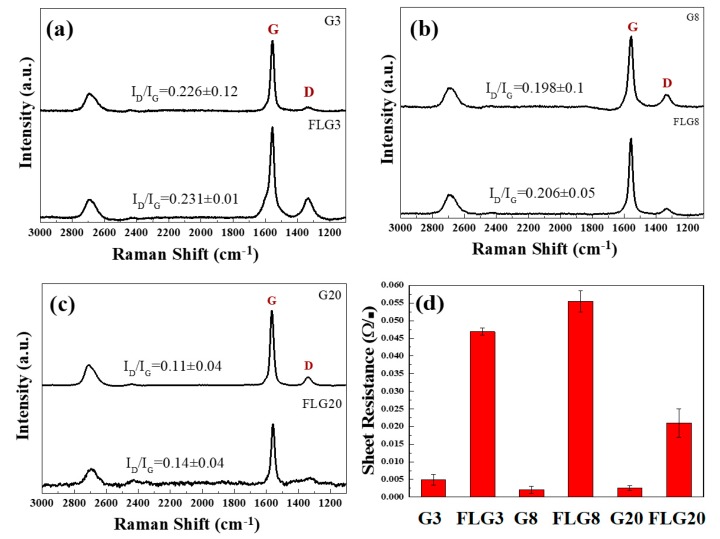
Raman spectra of (**a**) G3 and FLG3, (**b**) G8 and FLG8, and (**c**) G20 and FLG20, and (**d**) the sheet resistance of graphite and FLG.

Figure 4a–c show the Raman spectra of graphite and FLG. The main features in the Raman spectra of carbons are the so-called G and D peaks, which lie at around 1560 and 1360 cm^−1^, respectively, for visible excitation. The G peak is due to the doubly degenerate zone center E_2g_ mode, while the D peak is a breathing mode of *κ*-point phonons of A_1g_ symmetry [41]. The intensity ratio (I_D_/I_G_) of the D peak to G peak of the G3, G8, and G20 was about 0.226, 0.198, and 0.11, respectively. However, for FLG3, FLG8, and FLG20 which were obtained by LTHPD, the I_D_/I_G_ was 0.231, 0.206, and 0.14, separately. The I_D_/I_G_ ratio increased because the stripping process created defects. The results of graphite and FLG for testing the four-point probe are shown in Figure 4d. The sheet resistance of FLG was higher than that of graphite because the defects of FLG were increased; however, FLG was bulkier for graphite, which was beneficial for manufacturing the ECAs. The sheet resistance of FLG20 was lower than that of FLG. Therefore, we used FLG20 as the foremost material to export and apply to ECAs.

**Figure 5:**
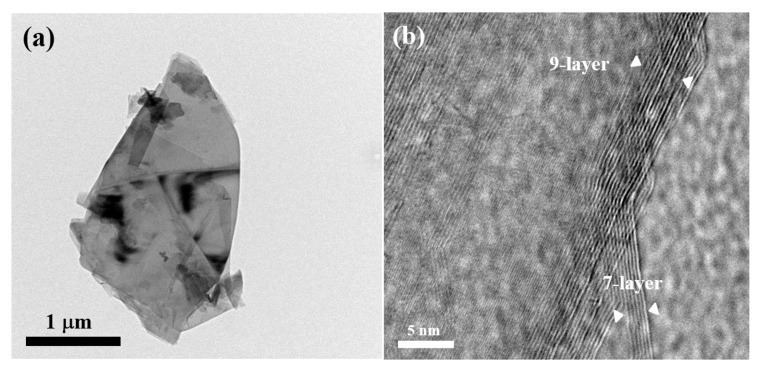
(**a**) Transmission electron microscopy (TEM) image of FLG20 and (**b**) high-resolution transmission electron microscopy (HRTEM) image of FLG20.

The morphology of FLG20 were observed by TEM and HRTEM. Figure 5a shows that FLG20 appeared as a micro-size transparent sheet structure with a smooth surface and wrinkled pattern on the edge, which was typical of graphene. An HRTEM analysis of folding at the edges of sheets gave the number of layers by direct visualization as Figure 5b, and it was clear that the number of layers of FLG20 was about 7~10 layers.

**Figure 6:**
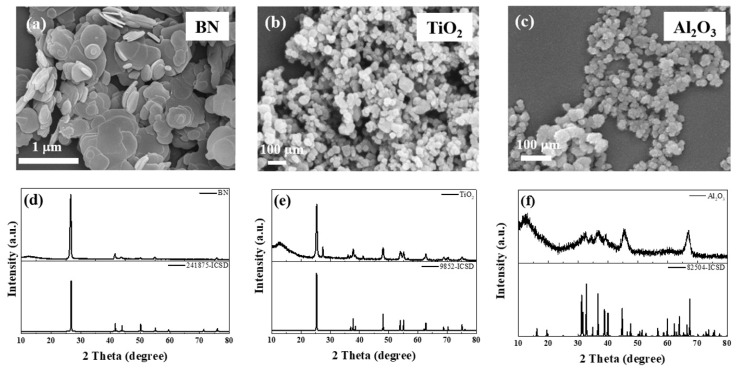
SEM images of (**a**) boron nitride (BN), (**b**) titanium dioxide (TiO_2_), and (**c**) alumina (Al_2_O_3_); XRD patterns of (**d**) BN, (**e**) TiO_2_, and (**f**) Al_2_O_3_.

Figure 6a–c show SEM images of BN, TiO_2_, and Al_2_O_3_, respectively. The morphology of BN was two-dimensional and its lateral size was in the range of 30–50 nm, as shown in Figure 6a. TiO_2_ and Al_2_O_3_ are granular materials, and had particle sizes of 20–30 and 10–15 nm, respectively (Figure 6b,c). In the study, we used these nanoparticles as nano fillers in ECA_S_, with the purpose of preventing the agglomeration of graphene lamellae and propping up the graphene to form a three-dimensional structure to increase conductive channels. The XRD patterns of BN, TiO_2_, and Al_2_O_3_ are shown in Figure 6d–f. All these diffraction peaks match well with the standard values and are in agreement with the hexagonal structure of the Bragg positions in ICSD-241875, ICSD-9852, and ICSD-66559, respectively. In Figure 6d, the XRD patterns had a peak at 23.5°, showing that the type of TiO_2_ was retile, and the Al_2_O_3_ was γ-phase Al_2_O_3_, as shown in Figure 6f.

**Figure 7:**
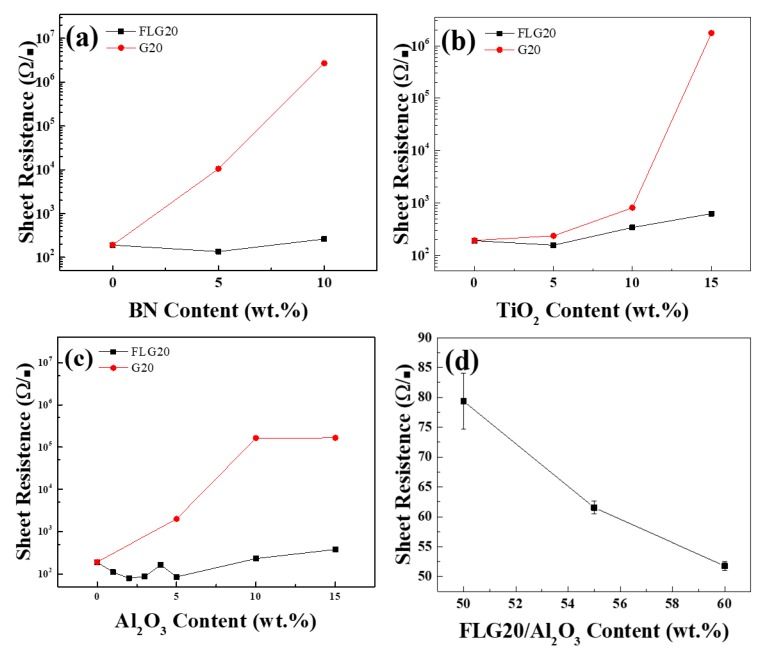
Sheet resistance of electrically conductive adhesives (ECAs) filled with different proportions of (**a**) BN, (**b**) TiO_2_, and (**c**) Al_2_O_3_. (**d**) Different FLG20/Al_2_O_3_ solid contents.

Figure 7a–c and Table S1 compare the sheet resistance of ECAs with G20 and FLG20 for different nano filler mass ratios. The blank EACs manufactured by G20 and FLG20 had a sheet resistance of 192 Ω/■ and 190 Ω/■, respectively. When the mass ratio of nano fillers to G20 increased, the sheet resistance of ECAs increased; however, the mass ratio of nano fillers to FLG20 increased, and the sheet resistance of ECAs decreased first and then increased. This result shows that FLG20 had better consequences because FLG20 was fluffier, so it generated a continuous conductive pathway easily. When the mass ratio was 95:5 (FLG20/nano fillers), all the sheet resistances reached a minimum value of 135 Ω/■, 156 Ω/■, and 85.7 Ω/■, respectively, which were 20%~56% lower than the blank. This result can be explained by the fact that the content of nano fillers was too high, so the nano fillers could not disperse raggedly, resulting in an increase in the sheet resistance. The effect of FLG20/Al_2_O_3_ was the best, so we continued to explore the ratios of 99:1, 98:2, 97:3, and 96:4, as shown in Figure 7c. When 2 wt.% Al_2_O_3_ was added, the sheet resistance reached a minimum value of 79.3 Ω/■, which was 60% lower than the blank. With the FLG20 to Al_2_O_3_ mass ratio of 98:2, Al_2_O_3_ prevented the agglomeration of FLG20 and propped it up to form more electrically conductive networks. As shown in Figure 7d, we increased the solid content to 55% and 60%, and the sheet resistance reduced from 79.3 Ω/■ to 51.8 Ω/■, which was a reduction of 73% compared to without adding any nano fillers. This result shows that increasing the solid content obviously decreased the sheet resistance.

**Figure 8:**
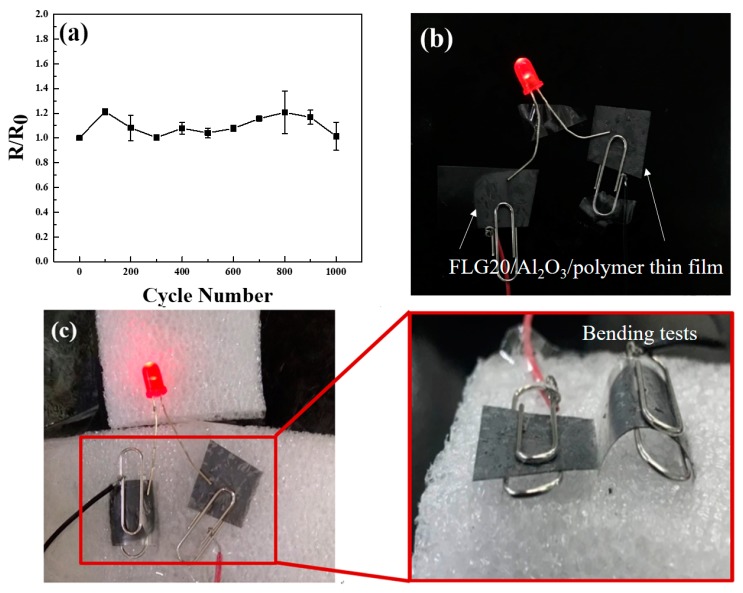
(**a**) The resistance increase ratio (R/R_0_) of FLG20/Al_2_O_3_/polymer thin film on PET under a bending performance test. After connecting the FLG20/ Al_2_O_3_/polymer composite to an LED, the light remained bright at various degrees of bending: (**b**) flat and (**c**) bending.

The FLG20/Al_2_O_3_ composite also exhibits a great flexibility and mechanical strength after being coated on flexible plastic sheets. The electrical resistance of the FLG20/Al_2_O_3_ thin film on the PET film was revealed by the bending test. As shown in Figure 8a, there was a fairly small amount of variation after thousands of bending cycles, with an R/R_0_ value retention value of 98%. This great mechanical strength makes the FLG20/Al_2_O_3_ a great conductive adhesive for flexible electronic applications. For demonstration, two pieces of printed FLG20/Al_2_O_3_ thin films on the PET film were connected to an LED. As shown in Figure 8c, the PET film with slight curvature could remain bright, showing that the EACs had a flexible property.

**Figure 9:**
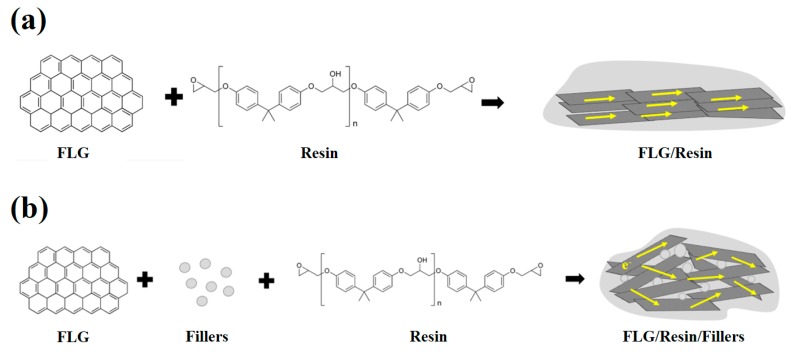
The before and after schematic diagram of the filler dispersion state. (**a**) Graphene composite with no filler, and (**b**) graphene composite with nano fillers.

Figure 9 is a schematic diagram of the situation before and after adding nano fillers. In Figure 9a, since graphene is a two-dimensional material, a large number of graphene may generate much restacking without adding nano fillers, causing the electrical conductivity to decrease. Therefore, adding different kinds of nano fillers to prevent the agglomeration of graphene lamellae and prop up the graphene to form a three-dimensional structure to increase the conductive channels is beneficial for decreasing the resistance, as shown in Figure 9b. In the study, adding nano fillers to graphene sheets could effectively reduce the resistance value.

## 4. Conclusions

We succeeded in delaminating artificial graphite and natural graphite by jet cavitation to prepare few-layered graphene (FLG3, FLG8, and FLG20). The structure and morphology of few-layered graphene had a two-dimensional structure and few-layered graphene was composed of thinner sheets and smaller sizes than graphite. We used G20 and FLG20 as the foremost material to apply to ECAs with different nano fillers (BN, TiO_2_, and Al_2_O_3_) to prevent graphene stacking and generate a continuous conductive pathway. The results indicated that the solid content was 60% and the best condition was adding 2 wt.% Al_2_O_3_, for which the sheet resistance value was 51.8 Ω/■. The electrically conductive resin remained nearly the same after one thousand bending cycles. These results indicate that the formulated FLG/Al_2_O_3_/polymer composite adhesives have a great potential in conductive adhesives for flexible display applications.

## Supplementary Materials

The following are available online at https://www.mdpi.com/2073-4360/11/10/1713/s1, Figure S1: SEM images of (a) KS-6, (b) 8 μm, (c) MoKS-6, (d) Mo8 μm, (e) BN and (f) TiO_2_, Figure S2: Raman spectra of (a) KS-6, (b) 8 μm, (c) MoKS-6 and (d) Mo8 μm, Figure S3: Sheet resistance of MoKS-6, Mo8 μm and MoKS-44, Figure S4: KS-44 and MoKS-44 composite with various content (a) BN and (b) TiO_2_, Table S1: KS-44 and MoKS-44 composite with various content BN and TiO_2_.
