# Supplementary material for: Structural Design of Three-Dimensional Graphene/Nano Filler (Al2O3, BN, or TiO2) Resins and Their Application to Electrically Conductive Adhesives
Source: Polymers (Basel). 2019 Oct 18;11(10):1713. doi: 10.3390/polym11101713 (PMC6835405; doi:10.3390/polym11101713)
Supplement: Supplementary file 1 [file polymers-11-01713-s001.pdf]

Supplementary Martials

Structural Design of Three-Dimensional Graphene/Nano fillers (Al_2O_3, BN or TiO_2) Resins and Its Application on Electrically Conductive Adhesives

Chia-Hsin Zhang [1], Chia-Hung Huang [2] and Wei-Ren Liu [1,*]

[1] Department of Chemical Engineering, R&D Center for Membrane Technology, Center for Circular Economy, Chung-Yuan Christian University, Chungli 32023, Taiwan; cindy560559@gmail.com

[2] Metal Industries Research and Development Centre, Kaohsiung 81160, Taiwan

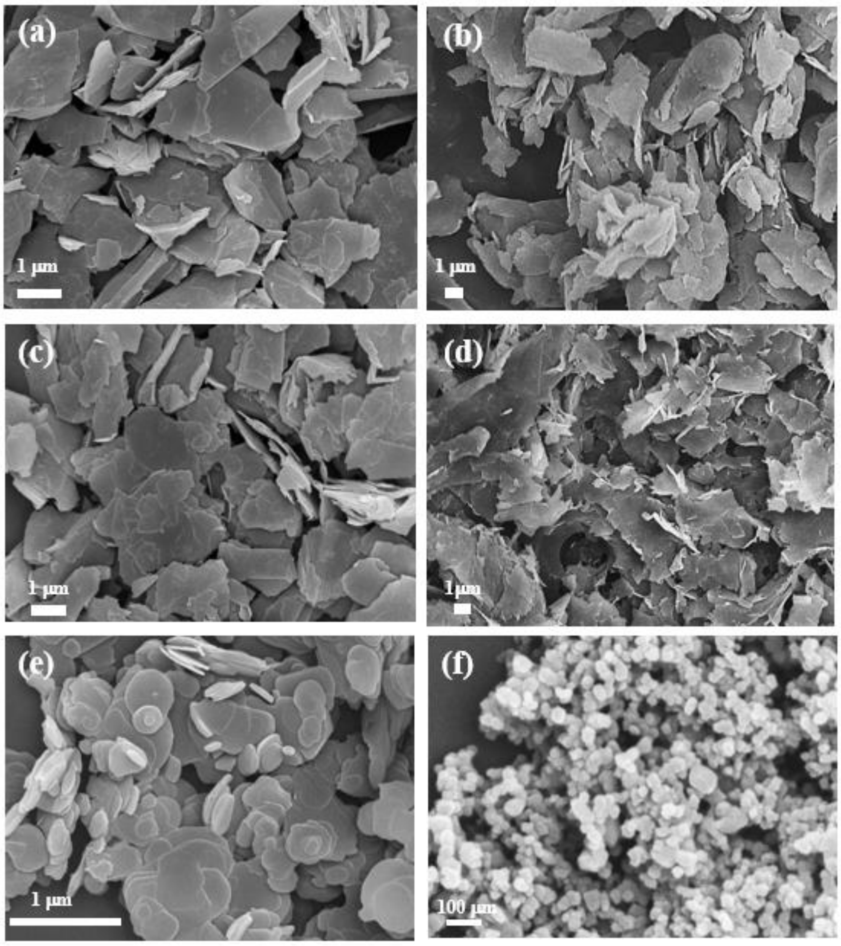

Fig. S1 SEM images of (a) KS-6, (b) 8 μm, (c) MoKS-6, (d) Mo8 μm, (e) BN and (f) TiO_2.

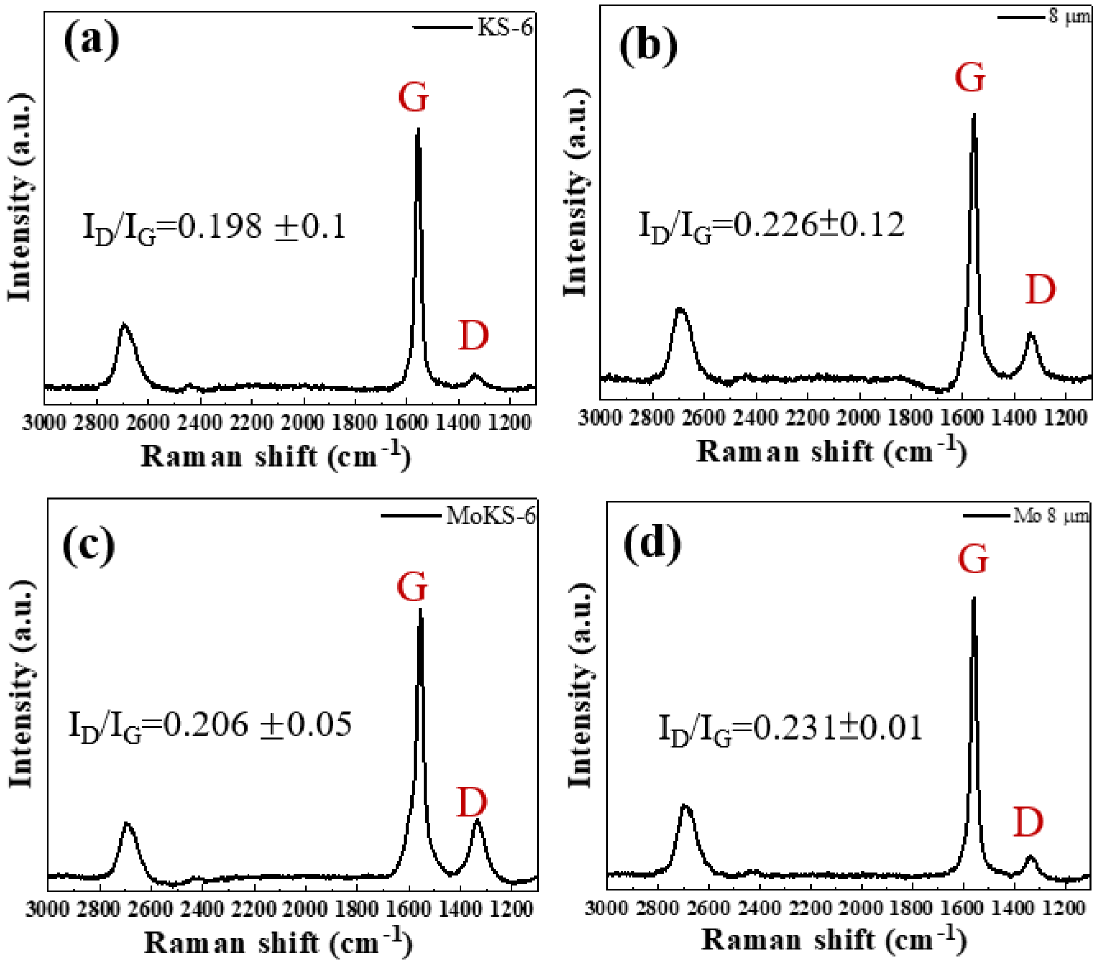

Fig. S2 Raman spectra of (a) KS-6, (b) 8 μm, (c) MoKS-6 and (d) Mo8 μm.

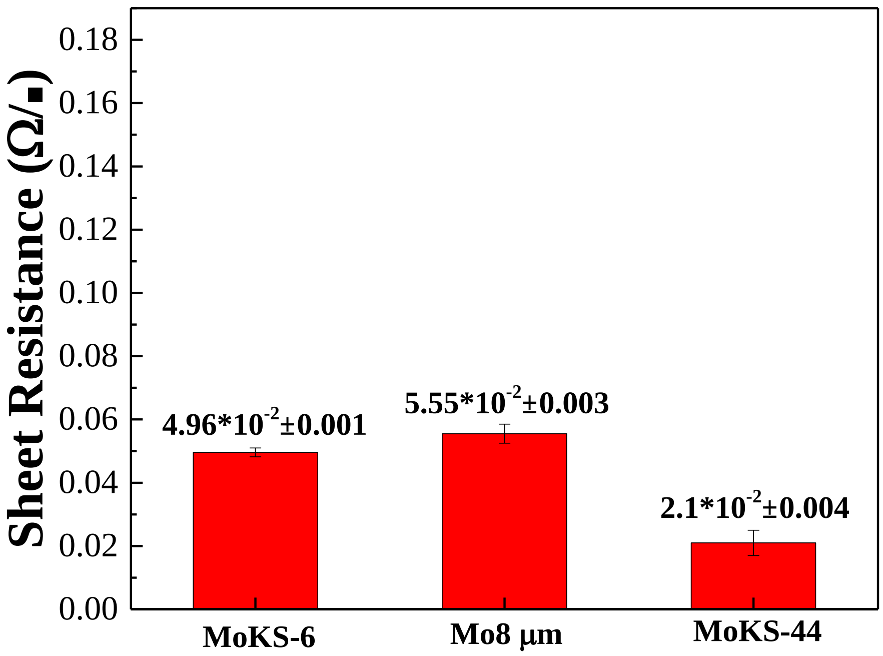

Fig. S3 Sheet resistance of MoKS-6, Mo8 μm and MoKS-44.

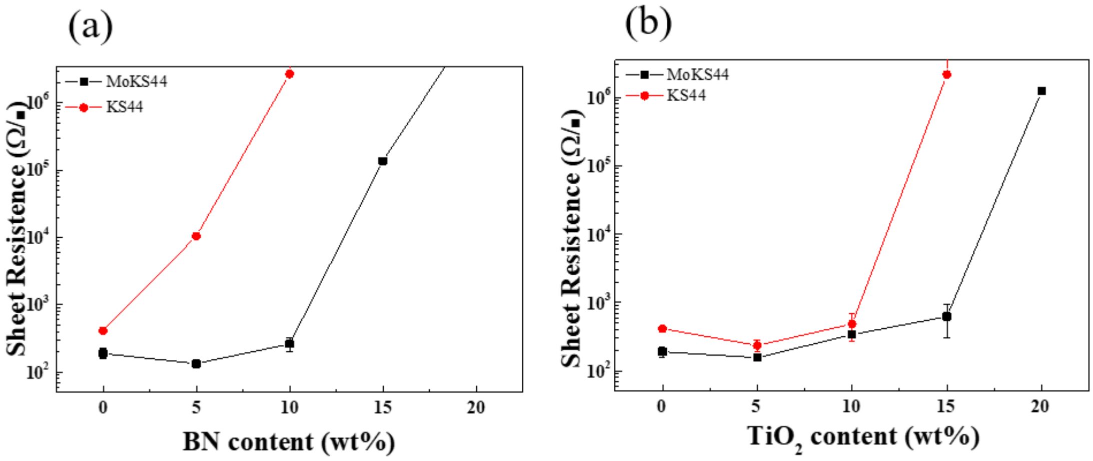

Fig. S4 KS-44 and MoKS-44 composite with various content (a) BN and (b) TiO_2.

Table. S1 KS-44 and MoKS-44 composite with various content BN and TiO_2.

	100:0 (Ω/■)	95:5 (Ω/■)	90:10 (Ω/■)	85:15 (Ω/■)	80:20 (Ω/■)
KS-44:TiO_2	414±39.4	235±47.8	486±210.7	$2.15*10^6±3.5*10^4$	$3.23*10^{37}$
MoKS-44:TiO_2	190±33.2	156±16.7	339±29.4	622±312.9	$1.23*10^6±7.54*10^4$
KS-44:BN	414±39.4	$1.05*10^4±47.8$	$2.69*10^6±779.4$	$3.23*10^{37}$	$3.23*10^{37}$
MoKS-44:BN	190±33.2	135±19.77	262±57.02	$1.37*10^5±2.29*10^3$	$1.72*10^7±4.5*10^5$
